# Research trends and hotspots in clinical trials of migraine in the past 20 years: bibliometric analysis

**DOI:** 10.3389/fneur.2024.1430138

**Published:** 2024-10-25

**Authors:** Xiaoxin Wang, Yan Sun, Yuan Zhang, Zhaohui Zhi, Shilin Wang, Jiaohui Li, Yingzhe Sun, Yuanzheng Sun

**Affiliations:** ^1^Heilongjiang University of Chinese Medicine, Harbin, China; ^2^Department of Medical Examination, Second Affiliated Hospital of Heilongjiang University of Chinese Medicine, Harbin, China; ^3^Department of Acupuncture, Second Affiliated Hospital of Heilongjiang University of Chinese Medicine, Harbin, China

**Keywords:** migraine, clinical trials, bibliometric analysis, CiteSpace, VOSviewer, CGRP, precision medicine

## Abstract

**Background:**

Migraine is a widespread, recurrent primary headache disorder primarily characterized by severe pulsatile headache, typically on one or both sides. It is often accompanied by nausea, vomiting, and hypersensitivity to sound and light. Despite the availability of multiple drugs for migraine management, the condition often becomes chronic due to untimely or irrational drug use, significantly distressing patients and increasing the burden on families and society. Over the past two decades, numerous clinical studies on migraine have been published. This study aimed to provide a comprehensive summary of the current status and trends of migraine clinical trials through bibliometric analysis.

**Methods:**

We used visual network tools such as CiteSpace and VOSviewer to perform a knowledge graph analysis of publications related to migraine clinical trials extracted from the WoSCC.

**Results:**

This study analyzed 1,129 articles published in 389 journals from 61 countries. The number of publications on migraine clinical trials has steadily increased from 2004 to 2023. The United States and Albert Einstein College of Medicine are the leading countries and institutions in this field, respectively. Richard B. Lipton is the most prolific author, making significant contributions to the research. The journal Headache has the highest number of publications and citations in this area. Keywords such as “efficacy,” “RCT,” “CGRP,” “prophylaxis,” “disability,” “depression,” “questionnaire,” and “real-world effectiveness” received significant attention.

**Conclusion:**

This study identified reliable research hotspots and provided directions for clinicians. The treatment of migraine continues to be challenging. Future trends may include continued growth in migraine classification, risk factor analysis, and comorbidity studies. Research on CGRP and epigenetics will advance the progress of precision medicine in the migraine field.

## 1 Introduction

Migraine is a common neurological disorder characterized by moderate or severe headache attacks lasting 4–72 h and is considered the most common clinical symptom (1, 2). Approximately 1 billion people worldwide suffer from migraine, with an estimated one-year prevalence of up to 15% in adults (1). Migraine is the second most disabling neurological disorder and often occurs alongside depression, epilepsy, and other diseases, with significant negative effects on the economy and personal quality of life (3). Currently, the mechanisms underlying migraine attacks and maintenance are not fully understood. Studies suggest that different mechanisms affect migraine attacks in different stages. Cortical spreading depression may better explain the presence of migraine aura, and variations in the trigeminovascular system and its radiation in brain regions reveal the characteristic distribution of headache during migraine attacks, including areas such as the eyes and periorbital region, forehead and temples, as well as pain radiating to the occipital and neck regions (1, 2). For migraine patients without contraindications, migraine-specific drugs are recommeded first-line treatment for controlling moderate or severe migraine attacks (3). However, for patients with frequent migraine attacks, drug overuse often occurs, and the use of triptans, ergotamines, or combination analgesics should be limited to no more than 10 days per month (4). Recent studies have focused on calcitonin gene related peptide (CGRP) receptor antagonists and monoclonal antibodies as novel targeted therapeutic agents (5, 6). Additionally, a possible predisposing factor, hypomagnesemia, has been identified, and daily supplementation of 400–600 mg magnesium citrate has been recommended with a quality of evidence graded as B (7).

Clinical trials are systematic studies conducted on humans, including both patients and healthy volunteers, to assess the effectiveness and safety of interventions. Randomized controlled trials (RCT) are considered the most rigorous and reliable research method for establishing causal associations between interventions and results, thus providing high-quality evidence for clinical decision-making (8). Nonetheless, the temporal, resource, and financial expenditures associated with clinical observation are significant. In the last 20 years, despite extensive scientific evidence endorsing various pharmaceutical treatments, physical interventions, and combination therapy for migraine management and long-term prevention, some problems persist unsolved (9, 10). Ongoing comprehensive clinical research will yield meaningful and reliable conclusions grounded in comprehending existing clinical studies. Due to reliance on manual literature searches, researchers appear constrained in their ability to identify and summarize high-impact publications, research hotspots, and prospective trends. Consequently, a novel and expedited method for organizing and extracting data from the literature, bibliometric analysis, can assist in identifying over-researched and overlooked topics in clinical migraine research, thereby enhancing comprehension of high-quality primary evidence and its implications for the field.

Bibliometric analysis employs mathematical and statistical methods to quantitatively analyze specific areas of literature. It can accurately identify the most influential researchers, manuscripts, journals, and institutions and discern significant research hotspots and trends. It serves as a tool to help researchers locate relevant papers, identify potential collaborators, find suitable journals for publication, and identify gaps in the literature that could be addressed (11). A systematic bibliometric analysis of clinical trials in the migraine treatment domain still needs to be included despite many reviews on the topic. Therefore, the study sought to examine the literature on clinical trials for migraine treatment during the past two decades to assist physicians in comprehending current research trends and advancements in this domain, hence fostering evidence-based practice for migraine management.

## 2 Methods

### 2.1 Data sources and search strategies

**Figure 1:**
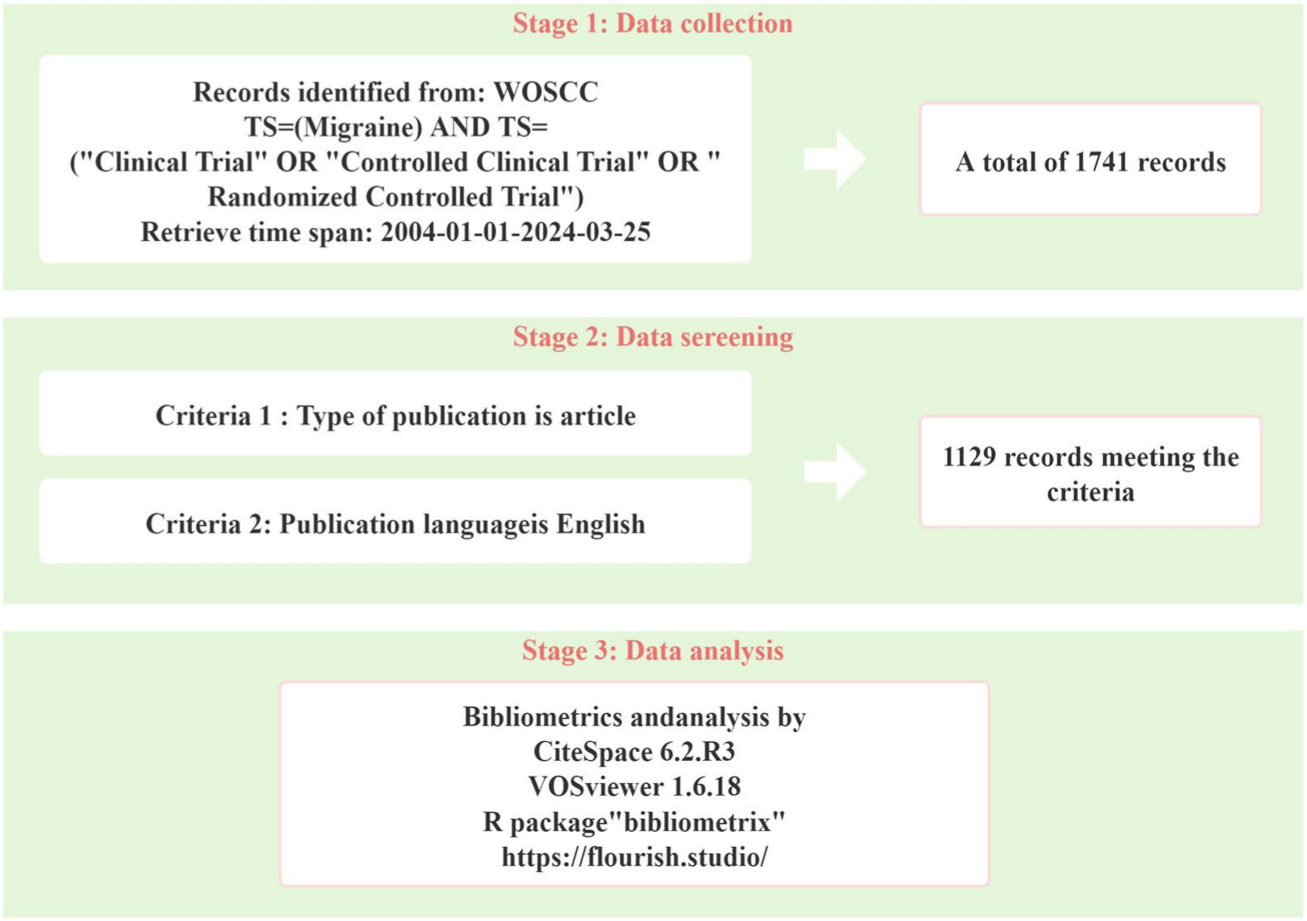
Flow chart of the literature retrieval process.

The study collected articles from the Web of Science Core Collection (WoSCC) database, which comprises over 12,000 international academic journals and is recognized as a highly comprehensive and reputable database (12). The search strategy employed was as follows: TS = (migraine) AND TS = (“clinical trial” OR “randomized controlled trial” OR “controlled clinical trial”). The search period included January 1, 2004, to March 25, 2024. Additionally, the search was limited to articles published in English and included only articles (Figure 1 illustrates the data collection process).

### 2.2 Data extraction and analysis

For visual analysis, CiteSpace (version 6.2. R 3) and VOSviewer (version 1.6.18). CiteSpace was used to analyze clustering, timeline trends, major reference bursts, and keyword bursts, with the aim of exploring the current research landscape, hotspots, and trends in the field. The nodes indicate different references or keywords, and the size and color of the nodes are determined by the frequency of citations and the year of publication, respectively. The annual citations for each reference or keyword are presented as citation tree annual rings. The most recent citation corresponds to the innermost ring. The network is decomposed into clusters based on the strength of the same cited links to the references or keywords. Clusters are numbered in descending order of their size. The largest one is numbered #0, then #1, and so on. VOSviewer was used to analyze cooperative networks among countries/regions, institutions, journals, and authors and to construct keyword co-occurrence networks. Circles and labels constitute a node, where the circle’s size is positively correlated with the frequency of occurrence of a country or institution, and the thickness of the connecting lines is positively correlated with the strength of the relationship between the countries or institutions, with distinct colors representing different countries or institutions, respectively. Furthermore, the R package “bibliometrix” and the website https://flourish.studio/were employed to conduct supplementary analyses, including mapping intercountry/region partnerships and creating alluvial flow diagrams for countries/regions, institutions, and journals.

### 2.3 Research ethics

Ethical approval was needed as this study did not include patients or animals.

## 3 Results

### 3.1 Annual growth trend of publications

**Figure 2:**
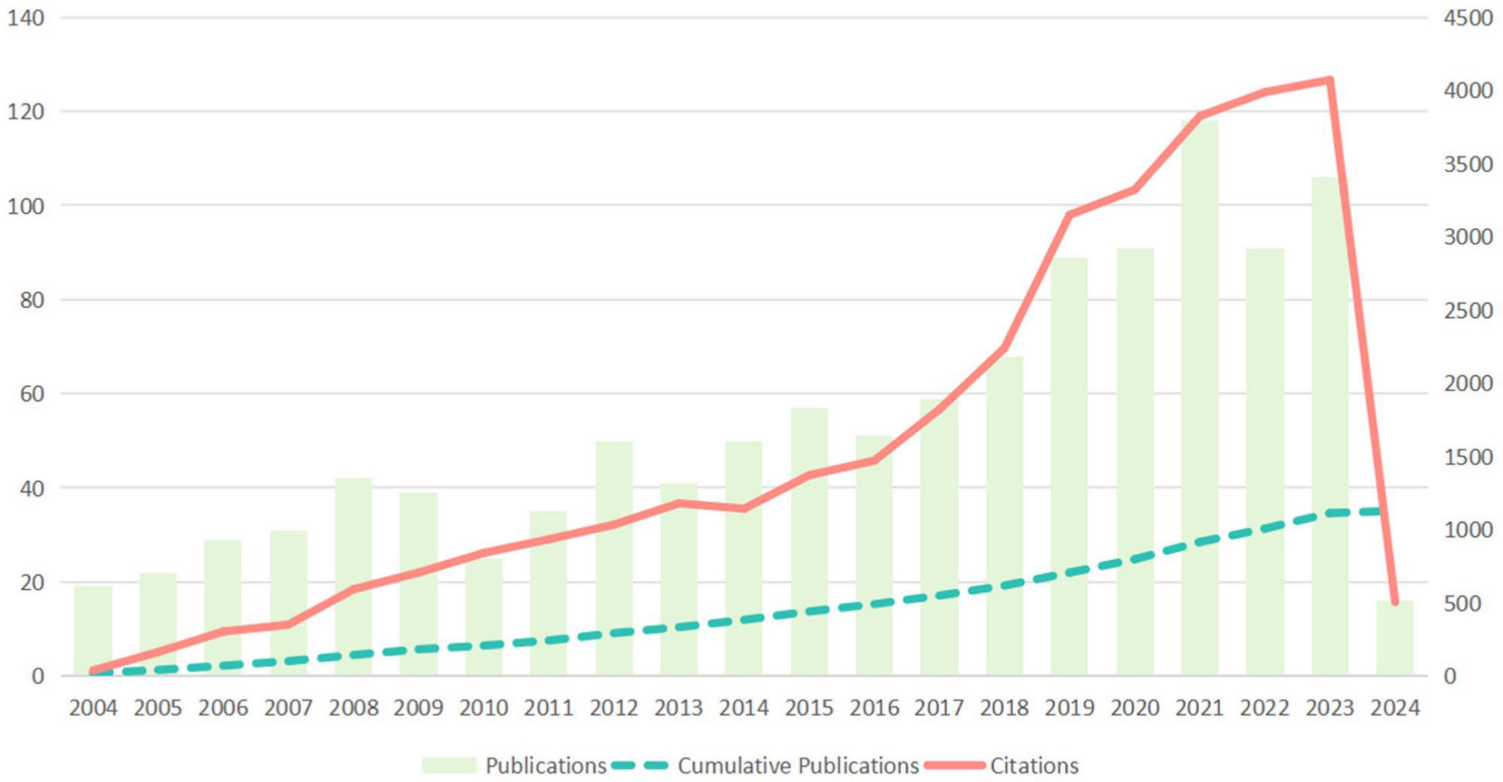
Trends in publications and citations per year from 2004 to 2024.

Between 2004 and 2024, a total of 1,129 complete publications on migraine clinical trials that met the screening criteria were obtained from the WOSCC database. From 2004 to 2023, the number of publications consistently increased. In the last 5 years, the number of publications stayed above 80 articles per year. The highest number of publications was in 2021, with a total of 118 articles published and 3,825 citations (Figure 2). The papers analyzed in this study received a total of 33,049 citations, with an average citation frequency of 29.27 and an H-index of 82 in the academic sector.

### 3.2 Analysis of countries/regions and institutions

**Figure 3:**
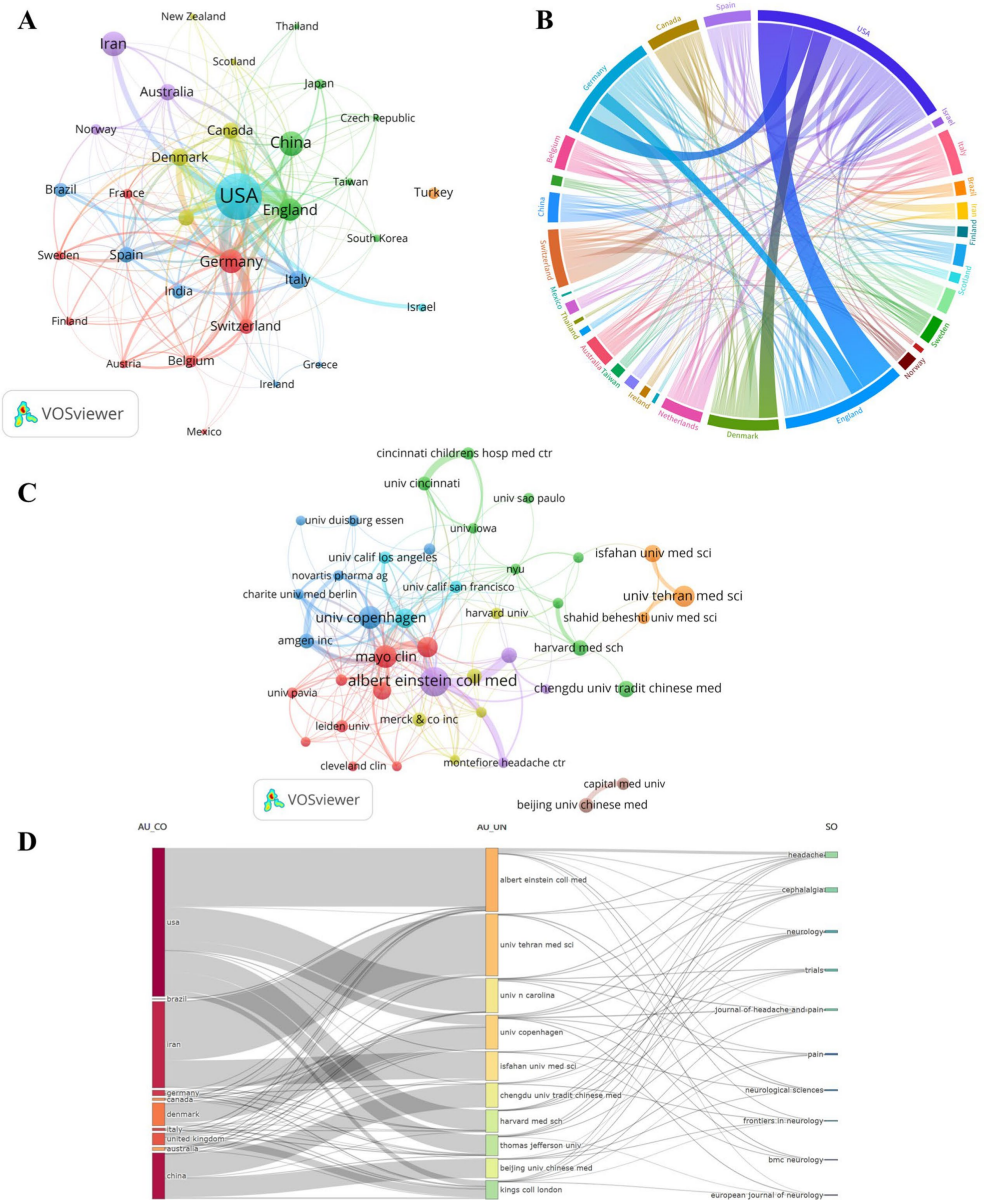
Countries/regions and institutions. (A) The network of co-authorship among countries/regions. (B) Collaborative visualization maps across countries/regions. (C) Co-authorship networks between institutions. (D) Alluvial flow diagrams showing the associations between countries/regions, institutions and journals.

A total of 61 countries and 1,791 institutions participated in the 1,129 publications on migraine clinical trials. Table 1 among the top 10 countries with the highest productivity, the United States of America (USA) and China contributed more than half of the publications in the field of migraine clinical trials. The USA had the highest number of publications (NP = 489), accounting for 41.13% of the total in the top 10 countries/regions, followed by China (NP = 120, 10.09%). Unsurprisingly, the USA also had the highest number of citations (NC = 19,612), followed by Germany (NC = 5,791), whereas China ranked fifth (NC = 1,828). Denmark had the highest average number of citations (AC = 58.19), followed by England (AC = 48.25). Although the USA had significantly more publications than Denmark and England, its average citation count was relatively low. Figure 3A shows the coauthor network between the 32 countries/regions that had at least 5 publications. It is worth noting that the network is divided into 6 clusters, indicated by different colors, except for Turkey, which did not form any associations with other countries/regions. The USA is located in the cluster represented by the light blue color and had the largest number of cooperating entities (*n* = 30). The closest cooperation occurred between the USA and England, followed by collaborations between the USA and Germany, Denmark, and England and Germany, as shown in Figure 3B. The top five countries in terms of total link strength (TLS) were the USA (TLS = 296), England (TLS = 176), Germany (TLS = 154), Denmark (TLS = 100), and Canada (TLS = 74). Table 1 presents the top 10 most influential institutions in the field. The institution with the highest NP value was Albert Einstein Coll Med (NP = 60), followed by Mayo Clin (NP = 40) and Univ Copenhagen (NP = 40). The most significant AC scores were from Thomas Jefferson Univ (AC = 83.97), Mayo Clin (AC = 72.40), and Univ Copenhagen (AC = 69.38). Four out of the 10 institutions were from the USA, whereas two each were from Iran and China.

**Table 1:** Top 10 most productive countries/regions and institutions.

Variables	NP	NC	AC	TLS	H-Index or location
Countries/regions					
USA	489	19,612	40.11	296	68
China	120	1828	15.23	42	23
Iran	119	1,615	13.57	24	25
Germany	110	5,791	52.65	154	37
England	95	4,584	48.25	176	31
Italy	60	1766	29.43	64	21
Denmark	57	3,317	58.19	100	25
Canada	49	1,249	25.49	74	21
Australia	47	842	17.91	41	16
Netherlands	43	1,144	26.60	58	19
Institutions					
Albert Einstein Coll Med	60	2,406	40.10	80	USA
Mayo Clin	40	2,896	72.40	88	USA
Univ Copenhagen	40	2,775	69.38	65	Denmark
Univ Tehran Med Sci	34	632	18.59	15	Iran
Thomas Jefferson Univ	33	2,771	83.97	59	USA
Kings Coll London	31	1775	57.26	58	England
Eli Lilly & Co	27	842	31.19	23	USA
Isfahan Univ Med Sci	25	270	10.80	10	Iran
Chengdu Univ Tradit Chinese Med	24	764	31.83	3	China
Beijing Univ Tradit Chinese Med	21	346	16.48	11	China

Figure 3C illustrates the network of co-authorship between institutions, with a total of 43 institutions having at least 10 publications. The top five total link strengths were Mayo Clin (TLS = 88), Albert Einstein Coll Med (TLS = 80), Univ Copenhagen (TLS = 65), Thomas Jefferson Univ (TLS = 59), and Kings Coll London (TLS = 58). Figure 3D shows the interconnections between countries/regions, institutions, and journals in the context of clinical trials targeting migraine. This plot consisted of 10 items per group. Four institutions from the USA are included, namely, Albert Einstein Coll Med, Univ N Carolina, Harvard Med Sch, and Thomas Jefferson Univ. The Iranian institutions include Univ Tehran Med Sci and Isfahan Univ Med Sci. Additionally, China is represented by Chengdu Univ Tradit Chinese Med and Beijing Univ Tradit Chinese Med. Albert Einstein Coll Med is associated with seven target journals, including Headache, Cephalalgia, Neurology, Journal of Headache and Pain, Neurological Sciences, Frontiers in Neurology, and European Journal of Neurology. Univ Tehran Med Sci is connected to four target journals, namely, Headache, Cephalalgia, Neurological Sciences, and BMC Neurology. Chengdu Univ Tradit Chinese Med also connects to four target journals, which are Headache, Neurology, Trials, and Frontiers in Neurology. It is worth noting that a significant portion of cooperation between institutions from the USA, Iran, and China primarily occurs within the framework of their respective regions or institutions.

### 3.3 Analysis of journals and authors

**Figure 4:**
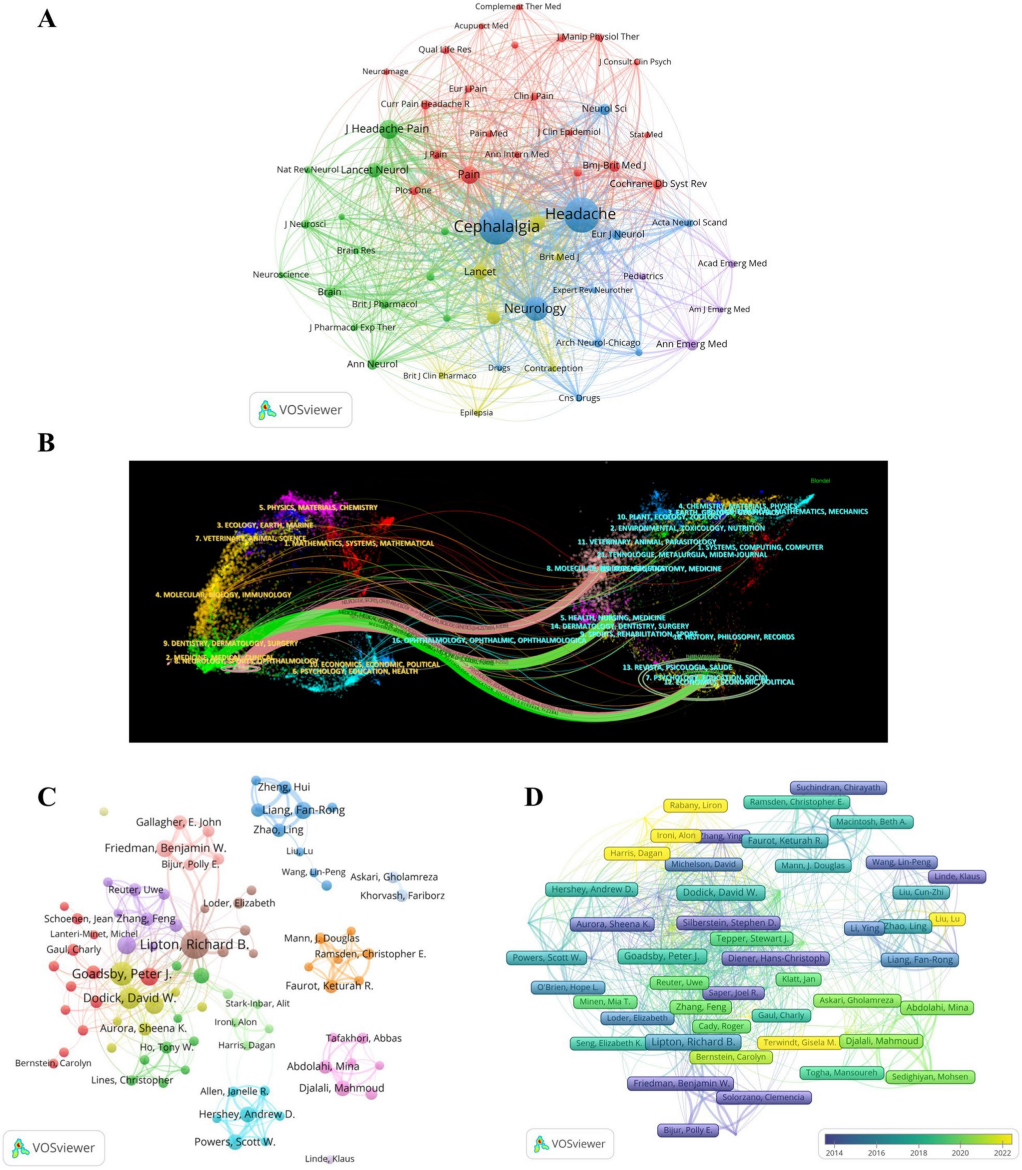
Journals and authors. (A) Network visualization for journal cocitation analysis. (B) Dual map overlay of journals. (C) Network visualization of coauthoring analysis. (D) Author citation analysis overlay visualization.

Articles on migraine clinical trials have been published in a total of 389 academic journals. Table 2 provides a list of the top 10 most influential academic journals that have published a significant number of guiding articles, accounting for 36.05% (407 out of 1,129) of the total publications. The journal “*Headache*” has published the most articles in the field and has received the most citations (NP = 131, NC = 5,014), followed by “*Cephalalgia*” (NP = 96, NC = 4,597). “Neurology” demonstrated a high level of citation influence, with an AC value of 107.40, nearly double that of the other journals. According to the Journal Citation Report, 60% of the top 10 journals were classified as Q1, whereas both Q2 and Q3 accounted for 20% each. Figure 4A shows that co-citation analysis conducted by VOSviewer identified a total of 57 articles with a minimum of 100 citations. The top three journals in terms of TLS were *Cephalalgia* (TLS = 169,569), *Headache* (TLS = 159,158), and *Neurology* (TLS = 73,565). The dual map overlay of the journals (Figure 4B) revealed 5 main citation pathways, with the most frequently covered domains being (1) neurology, sports, and ophthalmology and (2) medicine, medical, and clinical. The articles published in these journals mainly pertain to the following topics: (1) psychology, education, and social; (2) molecular biology and genetics; and (3) the effects of health, nursing, and medicine.

**Table 2:** Top 10 most influential academic journals and productive authors.

Variables	NP	NC	AC	IF and JCR or H-Index
Journals				
Headache	131	5,014	38.27	5.0, Q1
Cephalalgia	96	4,597	47.89	4.9, Q1
Journal Of Headache And Pain	45	1,369	30.42	7.4, Q1
Trials	29	290	10.00	2.5, Q3
Neurology	25	2,685	107.40	10.1, Q1
Frontiers In Neurology	21	83	3.95	3.4, Q2
Neurological Sciences	20	416	20.80	3.3, Q2
Pain	14	586	41.86	7.4, Q1
Bmc Neurology	13	148	11.38	2.6, Q3
European Journal Of Neurology	13	422	32.46	5.1, Q1
Authors				
Lipton, Richard B.	42	1956	46.57	25
Goadsby, Peter J.	31	2,234	72.06	19
Dodick, David W.	26	2,241	86.19	22
Ashina, Messoud	20	1,054	52.70	14
Silberstein, Stephen D.	19	2,152	113.26	11
Tepper, Stewart J.	18	488	27.11	11
Friedman, Benjamin W.	17	412	24.24	14
Liang, Fan-Rong	17	669	39.35	12
Ailani, Jessica	14	900	64.29	7
Djalali, Mahmoud	14	325	23.21	11

A total of 5,471 authors contributed to the publication of the 1,129 articles included in this analysis. Table 2 also presents the top 10 most productive authors in the field. Richard B. Lipton was the most prolific author, with 42 articles and 1,956 citations. This was followed by Peter J. Goadsby (NP = 31, NC = 2,234) and David W. Dodick (NP = 26, NC = 2,241). Co-authorship analysis using VOSviewer revealed a total of 89 authors who met the requirement of publishing at least 5 articles. Figure 4C displays the 14 clusters of authors, with each cluster annotated with a different color. The largest cluster (red cluster) consisted of 13 authors, with Stewart J. Tepper having the highest number of publications (NP = 18). The four clusters represented by Fan-Rong Liang (NP = 17), Keturah R. Faurot (NP = 11), Mahmoud Djalali (NP = 14), and Andrew D. Hershey (NP = 14) all showed isolated collaborations.

Furthermore, an analysis of author citation overlay visualization was conducted (Figure 4D). Klaus Linde was identified as the earliest author in the field of migraine clinical trials, whereas Richard B. Lipton stood out as the most influential author during the midterm period. Alit Stark-Inbar, Dagan Harris, and Alon Ironi were identified as emerging authors in the field. The top three authors based on TLS were Richard B. Lipton (TLS = 437), Peter J. Goadsby (TLS = 415), and David W. Dodick (TLS = 293).

### 3.4 Analysis of references

**Figure 5:**
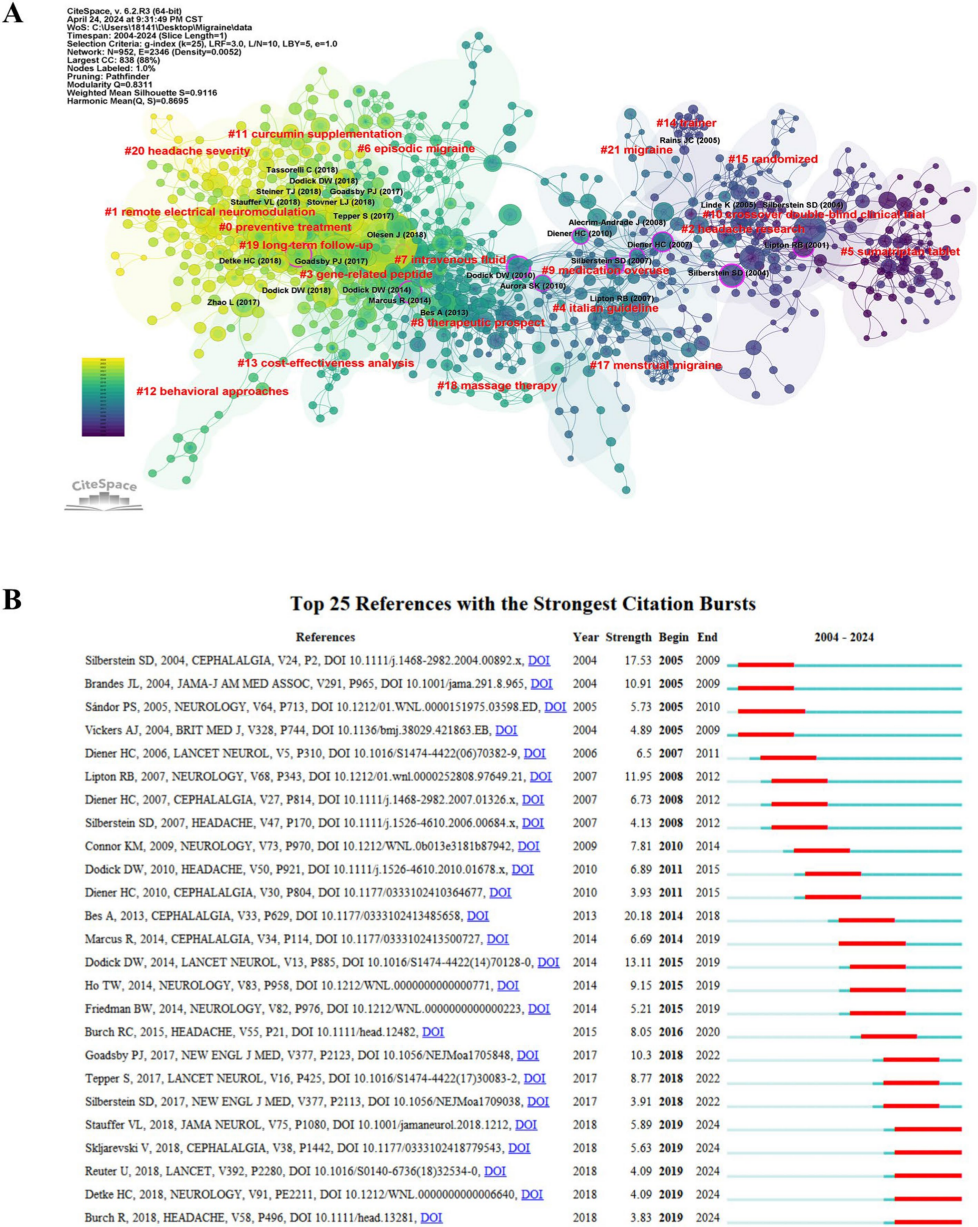
The references were analyzed with CiteSpace. (A) Clustering of references based on the similarity between references, including #0 preventive treatment and #1 lectrical neuromodulation. (B) The top 25 references with strong citation bursts. Red bars indicate high citations for the corresponding year.

Table 3 presents the top 10 most cited articles related to migraine clinical trials. The article titled “Headache Classification Committee of the International Headache Society (IHS) The International Classification of Headache Disorders, 3rd edition,” published in Cephalalgia in 2018, was the most referenced, with 142 citations. This paper provides a comprehensive classification and identification of migraine, offering a standardized foundation for subject selection in clinical trials (4). Two papers co-cited by another literature were reported to have a co-citation relationship. Co-citation cluster analysis objectively delineates the knowledge structure of a research domain, pinpointing seminal publications, foundational information, and emerging research horizons within the field. To further delineate the clusters of co-cited references, we employed the clustering feature in CiteSpace, utilizing the LLR method to extract nominal terms from the cited works’ titles, generating a network diagram (Figure 5A). The network has a high *Q*-value (*Q* = 0.8311) and (*S* = 0.9116), signifying it is a well-structured and highly credible clustering model. Dodick DW’s article “OnabotulinumatoxinA for treatment of chronic migration: pooled results from the double blind, randomized, placebo-controlled phases of the PREEMPT clinical program,” published in the journal Headache in 2010, demonstrates high centrality (13). It explores the preventive treatment of chronic migraine and suggests the efficacy of onabotulinum toxin A in reducing headache frequency and related disabilities, challenging the previous exclusion of chronic migraine patients from migraine prevention trials due to perceived disability and treatment resistance (14, 15). The network comprises 21 clusters, with the largest cluster (#0 preventive treatment) consisting of 108 members and a silhouette value of 0.862. The initial clusters were #5 sumatriptan tablets, #10 crossover double-blind clinical trials, and #2 headache research. Subsequent research progressed to the #9 medication overuse, #8 therapeutic prospects, and #7 intravenous fluid. In recent years, the connections between clusters have strengthened, particularly in areas such as #3 gene-related peptide, #0 preventive treatment, #1 remote electrical neuromodulation, #11 curcumin supplementation, and #20 headache severity. Figure 5B illustrates the top 25 references with the most significant citation bursts. The earliest four citation bursts began in 2005, whereas the most intense burst (20.18) occurred with BesA’s publication (16) in Cephalalgia in 2013, spanning from 2014 to 2018. The five most recent citation bursts occurred from 2019 to the present, with the highest burst (5.89) associated with Stauffer VL’s article “Evaluation of Galcanezumab for the Prevention of Episodic Migraine” (17), published in JAMA Neurol in 2018.

**Table 3:** Top 10 most cited articles regarding migraine clinical trials.

Rank	Title	Year	Author	Journal	Co-citations
1	Headache Classification Committee of the International Headache Society (IHS) The International Classification of Headache Disorders, 3rd edition	2018	Olesen J	Cephalalgia	142
2	The International Classification of Headache Disorders, 3rd edition (beta version)	2013	Olesen J	Cephalalgia	44
3	Global, regional, and national burden of migraine and tension-type headache, 1990–2016: a systematic analysis for the Global Burden of Disease Study 2016	2018	Stovner LJ	Lancet Neurology	43
4	A Controlled Trial of Erenumab for Episodic Migraine	2017	Goadsby PJ	The New England Journal of Medicine	34
5	Pathophysiology of Migraine: A Disorder of Sensory Processing	2017	Goadsby PJ	Physiological Reviews	32
6	Migraine pathophysiology and its clinical implications	2004	Silberstein SD	Cephalalgia	32
7	Safety and efficacy of LY2951742, a monoclonal antibody to calcitonin gene-related peptide, for the prevention of migraine: a phase 2, randomized, double-blind, placebo-controlled study	2014	Dodick DW	Lancet Neurology	30
8	Safety and efficacy of erenumab for preventive treatment of chronic migraine: a randomized, double-blind, placebo-controlled phase 2 trial	2017	Tepper S	Lancet Neurology	29
9	Migraine is first cause of disability in under 50s: will health politicians now take notice?	2018	Steiner TJ	Journal of Headache and Pain	25
10	ARISE: A Phase 3 randomized trial of erenumab for episodic migraine	2018	Dodick DW	Cephalalgia	24

### 3.5 Analysis of keywords

**Figure 6:**
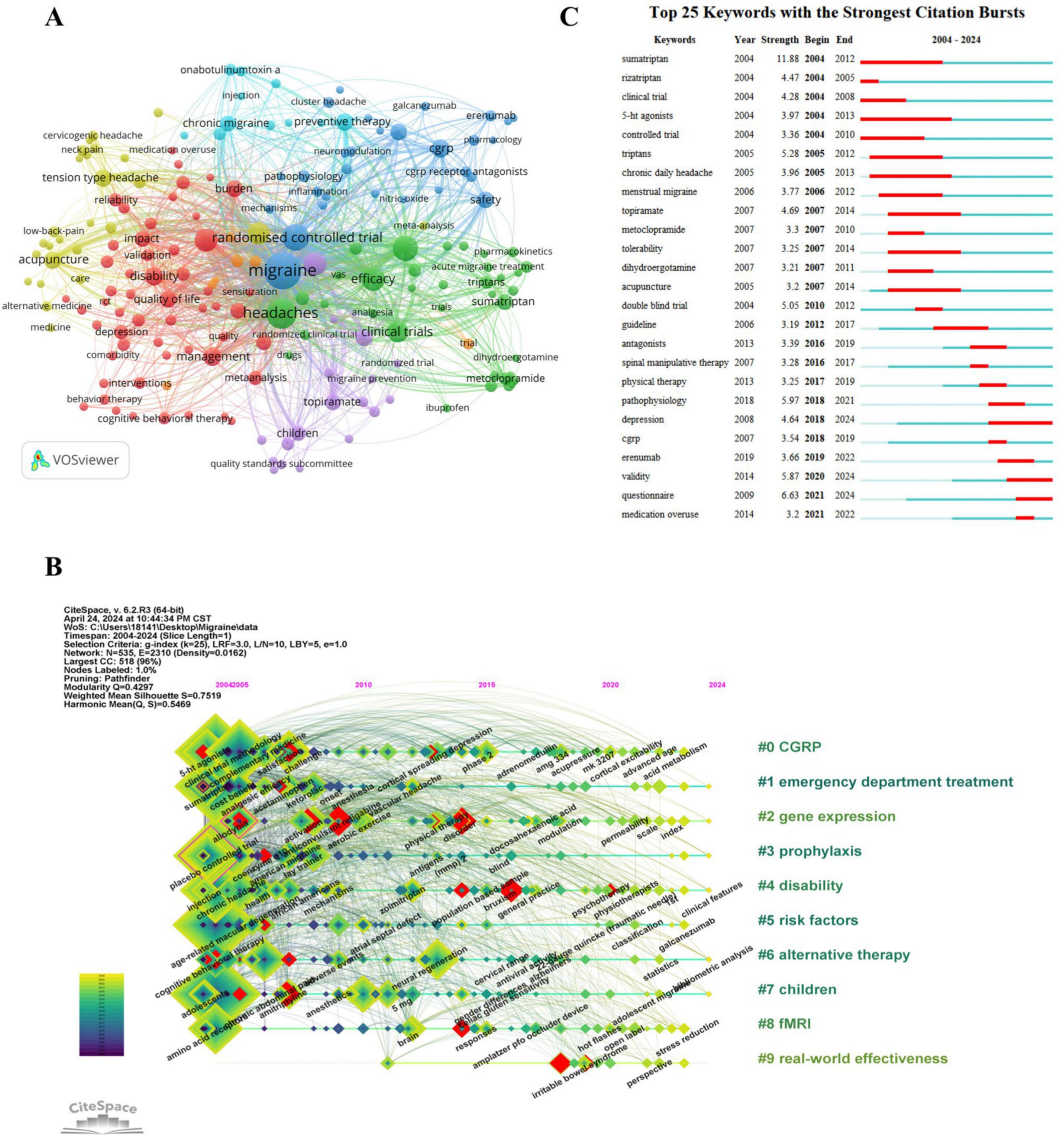
The references were analyzed with CiteSpace. (A) The keyword co-occurrence map. (B) A timeline view of keywords. (C) The top 25 keywords with the strongest citation bursts.

Generally, keywords in an article reflect the topic and research content, and changes in keywords can indicate the characteristics and trends of the publication. In this study, a keyword co-occurrence map was constructed using VOSviewer, as shown in Figure 6A, revealing 182 keywords that appeared at least 10 times. Table 4 provides a summary of the top 20 most frequently occurring keywords, excluding those related to the search topic such as “migraine,” “randomized controlled trial,” and “clinical trials.” Notably, keywords that appeared more than 100 times included “headaches,” “double-blind,” “efficacy,” “prophylaxis,” “prevalence,” “pain,” and “disability.” Among them, the number of TLSs of “headaches,” “double-blind,” “efficacy,” “prophylaxis,” and “prevalence” exceeded 1,000. CiteSpace has developed a keyword timeline viewer capable of clustering keywords. The clustering analysis of keywords uses the LLR technique, and noun terms are derived from the keywords to designate the clustering groupings. Considering the temporal aspect, it facilitates the analysis of the evolutionary trajectory of keywords across several clusters within migraine clinical trials and examines the study emphasis at each phase. To examine the evolutionary process of keywords in different clusters in the field of migraine clinical trials and explore research focuses at each stage, a timeline map of keywords was drawn using CiteSpace software, as depicted in Figure 6B. As of 2023, all 10 clusters are still in progress, with 6 clusters highlighting possible new research priorities in 2024. These clusters included #1 emergency department treatment, #2 gene expression, #3 prophylaxis, #4 disability, #6 alternative therapy, and #7 children. The largest cluster, #0 CGRP, revealed that the first keywords appearing in the field were “5-ht agonists” and “double-blind trial.” Moreover, gene expression in clusters #2 was associated with the most recent outbreaks, with “anti-inflammatory cytokines” being the most recent research direction. Last, #9 real-world effectiveness represents the most recent cluster, with the main keywords being “pathophysiology,” “erenumab,” and “comorbidity.” The evolution of keywords in the field of migraine clinical trials reflects an initial focus on symptomatic treatment in the acute phase of migraine, followed by increasing attention to refining the etiology, classification and staging treatment; incorporating complementary and alternative therapies; and emphasizing co-morbidities. Additionally, the burst of citations for keywords serves as an important indicator of research frontiers and model advancement. Figure 6C presents the top 25 keywords with the strongest citation bursts, with “sumatriptan” having the strongest bursts (11.88) from 2004 to 2011. Notably, “depression” (4.64), “validity” (5.87), and “questionnaire” (6.63) still experienced bursts.

**Table 4:** Top 20 most frequently occurring keywords.

Rank	Keyword	Occurrences	TLS	Rank	Keyword	Occurrences	TLS
1	migraine	686	3,825	11	management	99	601
2	headaches	401	2,489	12	CGRP	97	607
3	randomized controlled trial	277	1,649	13	acupuncture	93	601
4	double-blind	236	1,649	14	tension type headache	87	590
5	efficacy	195	1,365	15	preventive therapy	84	608
6	prophylaxis	187	1,192	16	placebo	82	530
7	prevalence	183	1,277	17	quality of life	81	547
8	pain	163	990	18	burden	78	573
9	clinical trials	154	939	19	episodic migraine	74	548
10	disability	101	716	20	safety	73	540

## 4 Discussion

### 4.1 Overview of migraine development clinical trials

Generally, the annual scientific output is a reliable indicator for measuring the research focus of scholars in a specific discipline (18). Over the past 20 years, the annual number of publications in the field of migraine clinical trials has steadily increased. This trend can be divided into two phases: the gradual growth phase from 2004 to 2017 and the accelerated expansion phase from 2018 to 2023. The year 2018 is notably significant, possibly due to improved diagnosis and classification of headache diseases (4), the inclusion of CGRP in clinical research (17), and the recognition of migraine complications (19). The number of publications reached its peak in 2021 and has since remained at approximately 80 publications per year, indicating that migraine clinical trials continue to be an area of ongoing attention in the medical community.

From a national or regional perspective, the United States is the most prolific country in the field and has made the largest contribution to the NC and H indices. It is worth noting that whereas China ranks second in NP, its NC, AC, and H indices are relatively lower. On the other hand, Germany, despite having a similar number of publications as China, has contributed more to quality clinical research. This suggests a need for improvement in the quality of research in the field of migraine clinical trials in China. Among the top 10 institutions in terms of productivity, the top five are from the United States, Denmark, and England. Apart from Germany, these countries are also the largest contributors to the AC index. The USA, England, Germany, and Denmark have established significant collaboration networks in the field, whereas institutions from China and Iran tend to prefer regional or institutional collaboration. This highlights the dominance of the United States in global research collaborations on migraine clinical trials. Establishing effective and extensive cooperation exchanges is crucial for advancing research in the field. Journal analyses provide valuable references for researchers to select appropriate journals (18). An analysis of journals and co-cited journals can serve as a dependable reference source for scholars’ contributions. Figure 4B presents a dual-map overlay of journals, offering a visual picture of the distribution of particular academic journals, the evolution of citation trajectories, and the shift in research focus. Headache and Cephalalgia are the two journals that publish the greatest number of articles on migraine clinical trials and are among the most co-cited journals. These two journals have played a vital role in migraine research. Journals predominantly prefer topics such as migraine health management, genetics, and pathophysiological mechanisms, particularly within the realms of neurology and clinical medicine.

Author co-citation refers to a co-citation relationship centered on authors as the fundamental unit, facilitating the aggregation of numerous authors into groups based on citation relationships. This process allows for the identification of highly cited authors within a specific field and the recognition of influential scholars. Furthermore, by analyzing the author co-citation network and its clustering, one can discern the research topics of similar authors and uncover the predominant academic groups and scholarly concepts within the domain of scientific knowledge. The co-citation network of writers and its grouping can elucidate the study themes of analogous authors within a specific domain, so uncovering predominant academic factions and prevailing intellectual concepts in the realm of scientific knowledge. Analyzing and interpreting the principal academic concepts and perspectives of these prominent researchers, while also identifying their internal interconnections within the author co-citation network, can more effectively elucidate the key academic ideas in the subject of migraine and their evolution and transmission. From the author’s perspective, Dr. Richard B. Lipton has been the most prolific author in the field of migraine clinical trials over the past 20 years. As the director of the Albert Einstein College of Medicine Montefiore Headache Center, Dr. Lipton’s research team is dedicated to conducting epidemiological studies and clinical trials related to migraine. In recent years, the main focus has been on the therapeutic potential of gepant drugs, specifically atogepant (20) and Ubrogepant (21), for the treatment of migraine. Additionally, Dr. Lipton’s team is involved in the Migraine Clinical Outcome Assessment System (MiCOAS) project. This project aims to establish a standardized set of results and endpoints for patient-centered clinical trials on migraine. As part of this project, they have examined the impact of migraine on the daily functioning of individuals, identifying 66 key factors. The authors also highlighted the significant influence of socio-environmental factors on these properties, contributing to a better understanding of the burden of migraine and the efficacy of migraine treatments (22). They also summarized risk factors for migraine progression to guide healthcare providers in targeting protective measures (23). Furthermore, Dr. Lipton and his collaborators, Dr. Peter J. Goadsby and Dr. David W. Dodick, have maintained a close partnership. Their earliest joint publication, dating back to 2004, explored the use of oral tretinoin analogs as a management option for migraines (24). Their most recent collaborative study focused on the use of ubrogepant in various aspects of migraine treatment, including duration of treatment (25), efficacy in the prodromal phase (26), and efficacy in the acute phase (27). These studies have shown that Ubrogepant is an effective and well-tolerated treatment for migraines. In addition to these contributions, Professor Fang-Rong from China has brought acupuncture treatment for migraines to the international stage. His research has centered on developing standardized protocols for the treatment of migraines using acupuncture (28). He has also investigated the preventive role of acupuncture in migraines. Professor Fang-Rong’s work complements traditional migraine treatment methods and provides a foundation for future clinical trials on acupuncture (29).

### 4.2 Research hotspots and frontiers in migraine clinical trials

Among the top 10 cited references, two articles on the classification and diagnostic criteria of headache were published in 2013 (16) and 2018 (4), respectively. The use of the ICHD-3 as a diagnostic tool for headache has been recognized and clinically applied by experts in the headache and neuroscience industries in various countries. This recognition not only validates the findings in one country but also bridges the gap between clinical practice and evidence-based medicine. However, certain issues still require further research to improve ICHD, such as distinguishing between migraine with brainstem aura and vestibular migraine, understanding the basis for the existence of retinotropic migraine, and clarifying the definition of chronic migraine (30). In 2024, a new study on headache classification was conducted, highlighting the continued vitality of this discipline (31). The exploration of migraine epidemiology has also attracted the attention of scholars in various countries, as evidenced by the inclusion of two articles on this topic in the top 10 cited references. These articles investigated the burden of migraine disease from 1990 to 2019, revealing significant increases in migraine burden over time and substantial variations between countries. Furthermore, the study of risk factors, diagnostic approaches, and treatment regimens for migraine progression in different populations (especially young adults versus women) continues to be a subject of ongoing interest (32). The article titled “Pathophysiology of Migraine: A Disorder of Sensory Processing” by Goadsby, P.J., et al. was published in Physiological Reviews in 2017 and provides valuable insights into the complex physiopathological basis of migraine. It emphasizes the central role of the brain in triggering migraines and attributes migraine onset to changes or dysfunction in the brainstem and hypothalamic regions (33). In recent years, imaging-based studies on migraine physiopathology have gained popularity among researchers, aligning with the results of keyword clustering (#8 functional magnetic resonance imaging). Furthermore, the neuropeptide CGRP has emerged as a key factor in the pathophysiology of migraine. As Table 3 indicates, four articles focused on clinical trials studying the effects of CGRP-related agents on migraine (34–37). These studies demonstrate the excellent preventive effects and safety of CGRP receptor antagonists and monoclonal antibodies, supporting their future utilization and emphasizing the role of CGRP in migraine pathogenesis. Although these novel medications have brought about changes in migraine treatment protocols, there remains a significant unmet need for migraine patients, particularly those who qualify as resistant or refractory (38, 39). Extensive clinical data are still required to determine the appropriate duration of treatment with new drugs, predictors of response, and potential benefits of switching between combinations and classes of preventive medications, along with other preventive medications (40).

Furthermore, quantifying CGRP presents challenges. The feasibility of using serum CGRP as a biomarker for chronic migraine appears to be limited (41). A study revealed that tear CGRP levels are 140 times greater than those in plasma and that CGRP levels in the tears of migraine patients are elevated during the interictal period and altered by pharmacological interventions compared to those in healthy controls. However, tear CGRP levels are not specific in regard to headache frequency (42). Similar measurements of CGRP levels have been conducted in cerebrospinal fluid (43) and saliva (44). Alpuente A’s team discovered changes in salivary CGRP during different attack phases, along with correlations with headache frequency, depressive symptoms, and pharmacological interventions (44–46). This finding suggested that salivary CGRP may hold potential as a biomarker for migraine. Taken together, these findings underscore the ongoing importance of conducting in-depth research on CGRP.

Among the top 20 keywords, four keywords related to clinical trial methods were of significance: “randomized controlled trial,” “double-blind,” “clinical trials,” and “placebo.” These keywords play a crucial role in establishing a reliable, evidence-based foundation. Additionally, clinical observations regarding treatment outcomes for migraine patients have focused on effectiveness, safety, and improvements in quality of life. This observation aligns with the primary outcome metrics highlighted in the top 10 cited articles shown in Table 3. The second largest cluster in the keyword clustering is related to emergency department treatment, labeled as #1. Headache is a complex issue often encountered in emergency departments. Multiple studies on the number of people visiting the Emergency Department with headaches have indicated that migraine is the predominant cause of primary headaches (47, 48).

Nevertheless, there are notable variations in the diagnosis and treatment of migraines in the emergency department. Patients frequently endure prolonged periods in environments ill-suited for headache management, resulting in excessive testing and precautionary admissions (47). Furthermore, opioid utilization remains prevalent in emergency room settings (49), particularly among rural communities (50). Benjamin W Friedman et al.’s study presented level I evidence supporting the use of intravenous dexamethasone 4 mg in the emergency department to decrease migraine recurrences and achieve prolonged headache relief (51). The management of migraine in the emergency department deserves to be scrutinized.

Cluster #2 gene expression research has shown significant growth in citations. Research has been expanding to explore gene regulatory mechanisms in the development of migraines, in addition to studying inflammatory factor expression in the trigeminal vascular system (52). Epigenetic modifications such as DNA methylation, histone acetylation, and microRNA-mediated control appear to be beneficial for comprehending migraine, including predicting risk, recognizing illness progression, diagnosing and determining prognosis (53). Winsvold et al. (54) conducted a study comparing the methylation levels of CpG loci in 36 female headache patients who had transitioned from episodic to chronic headaches with 35 female headache patients who had not progressed to chronic headache. The findings indicated that DNA methylation of the regions studied by NPTX2 and SH2D5 genes may be a potential mechanism of headache chronicity. CALCA (55, 56), miR-34a-5p (57), and miR-382-5p (58) are involved in migraine attacks and treatment response. Epigenetic mechanisms are valuable for categorizing patients, molecular diagnostics, and applying precise medical treatments for migraine (54).

The #9 real-world effectiveness cluster represents a novel and evolving research paradigm in clinical research on migraine. A real-world study (RWS) is a methodology for concluding by collecting and analyzing real-world data in a clinical context (59). This approach has gained traction among clinicians in recent years, as reflected in the preference for exploring “real-world effectiveness” as a relatively novel trend in clinical research. A meta-analysis demonstrated strong agreement between RWS and RCT results, highlighting the potential of RWS to guide in the absence of evidence from double-blind clinical trials (60). Additionally, analyzing Table 4 in conjunction with the keyword outbreak analysis allows for the prediction that the keywords “prophylaxis,” “disability,” “depression,” “questionnaire,” and “validity” are likely to gain prominence in the future. Based on the comprehensive keyword analysis presented, it becomes evident that future research in the field of migraine clinical trials will continue to focus on preventive treatment of the disease through both RCTs and RWS, specifically emphasizing the further development and refinement of CGRP-related drugs. Researchers will also direct their attention toward evaluating the long-term effectiveness of treatment methods in alleviating patients’ clinical symptoms, reducing the disability rate associated with migraines, and enhancing overall quality of life. Additionally, future research efforts should address potential migraine-induced risk factors, comorbidities, and the appropriate application of questionnaires for migraine evaluation. Precision medicine will be a significant focus driving research in the field of migraines in the long run.

## 5 Limitations

The data used in this analysis were obtained solely from the WoSCC database, and only articles written in English were included. Furthermore, publications published after the search date were excluded from our analysis, potentially resulting in incomplete coverage and introducing a certain degree of lag, which may have biased the results.

## 6 Conclusion

To the best of our knowledge, this study represents the first bibliometric analysis of clinical trials in the context of migraine. The findings presented here provide valuable insights into the major countries, institutions, journals, and authors contributing to the field of migraine clinical trials. Moreover, this study elucidates the current research hotspots and directions within this domain. Given the heterogeneity in the mechanisms, etiology, and symptoms of migraines, the treatment of this condition remains challenging. Consequently, future studies are expected to continue flourishing in the areas of migraine classification, risk factors, and comorbidities. Research on CGRP and epigenetics will advance the progress of precision medicine in the migraine field.

## Data Availability

Publicly available datasets were analyzed in this study. This data can be found here: Web of Science Core Collection database.
